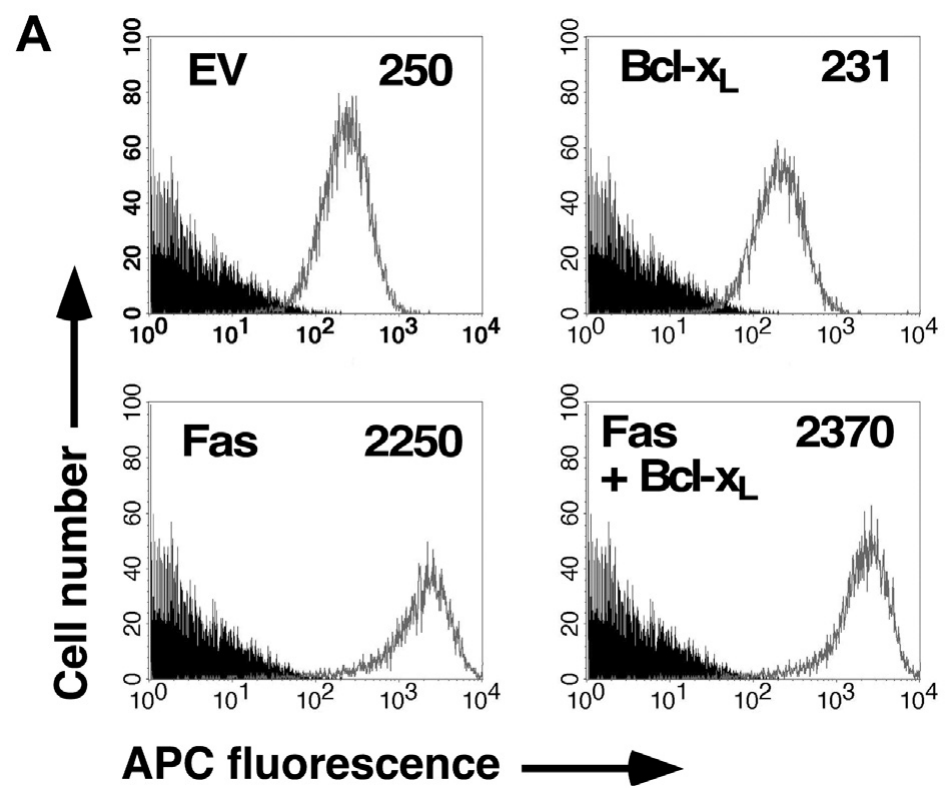# Correction: High cell surface death receptor expression determines type I versus type II signaling

**DOI:** 10.1016/j.jbc.2022.101980

**Published:** 2022-04-28

**Authors:** Xue Wei Meng, Kevin L. Peterson, Haiming Dai, Paula Schneider, Sun-Hee Lee, Jin-San Zhang, Alexander Koenig, Steve Bronk, Daniel D. Billadeau, Gregory J. Gores, Scott H. Kaufmann

In Figure 4*A* the Bcl-x_L_ 231 panel was incorrect.

The amended Figure 4*A* is presented below.

**Figure:**